# *Dens invaginatus* u odontoma dilatado, controversia de una definición

**DOI:** 10.21142/2523-2754-0902-2021-057

**Published:** 2021-06-21

**Authors:** Mayra Coria-Bello, Francisco Javier Morales-González

**Affiliations:** 1 Facultad de Odontología, Universidad Nacional Autónoma de México. Ciudad de México, México. mayracoria79@hotmail.com Universidad Nacional Autónoma de México Facultad de Odontología Universidad Nacional Autónoma de México Ciudad de México Mexico mayracoria79@hotmail.com; 2 Facultad de Odontología, Universidad Michoacana de San Nicolás de Hidalgo. Michoacán, México. pacomor_333@hotmail.com Universidad Michoacana de San Nicolás Hidalgo Facultad de Odontología Universidad Michoacana de San Nicolás de Hidalgo Michoacán Mexico pacomor_333@hotmail.com

**Keywords:** odontoma, dens in dente, diente invaginado, tomografía computarizada de haz cónico, dontoma, dens in dente, cone beam computed tomography

## Abstract

El diente invaginado (DI) es una alteración en el desarrollo del órgano dentario, el cual se produce a consecuencia de una invaginación del epitelio interno del órgano del esmalte, también conocido con otros nombres, entre ellos *dens in dent*. Tiene una serie de características clínicas, histológicas e imagenológicas debidamente descritas a través de la literatura. Su variante más extrema presenta una configuración muy compleja y, debido a la magnitud de la invaginación que presenta, se le denomina también odontoma dilatado, término que para algunos profesionales genera confusión.

Este término ha sido mencionado como sinónimo tanto de DI como, en algún momento, de una nueva variante de odontomas, si bien existe una diferencia entre ambos tipos: uno es una anomalía del desarrollo y el otro, un crecimiento de aspecto hamartomatoso. Sin embargo, la literatura sigue utilizando ambos términos para definir al DI, por lo que es importante conocer el origen y tener claro que la controversia está basada en referencias históricas y de costumbre.

## Abstract (en)

The invaginate tooth (IT) is an alteration in the development of the dental organ, which occurs as a consequence of invagination of the internal epithelium of the enamel organ. IT is also known as “dens in dente”, among other denominations, and presents a series of clinical, histological and imaging characteristics. The most extreme variant of IT has a very complex configuration, and the magnitude of the intussusception has led to it also being called dilated odontoma, being a term that generates confusion in some professionals.

While the term odontoma has been reported as a synonym for both IT as well as a new variant of odontomas, there is a difference betweene the two types: one is a developmental anomaly and the other is a growth with a hamartomatous appearance. However, the literature continues to use both terms to define IT, and thus, it is important to know the origin and be aware that the controversy is based on historical and customary references.

## INTRODUCCIÓN

El diente invaginado (DI), también conocido por otros sinónimos -entre ellos *dens in dent*-, tiene una serie de características clínicas, histológicas e imagenológicas debidamente descritas a través de la literatura. De acuerdo con su presentación se han postulado una serie de clasificaciones, siendo quizá la propuesta por Oehlers una de la más conocidas y referenciadas ^1^^-^^3^.

Dentro de esta clasificación se conoce que la variante más extrema (clase III a) presenta una manifestación muy compleja, debido a la magnitud de la invaginación que presenta, pues compromete la extensión de esmalte al interior de una estructura dentaria, revistiéndola como un ducto irregular que se prolonga hasta la zona apical o hasta encontrar una comunicación externa.

Uno de los sinónimos utilizados para esta alteración es odontoma dilatado (OD), el cual fue un término propuesto por Rushton (1936), quien, basado en aspectos clínicos y radiográficos, lo definió en su momento como una variante del odontoma. 

A partir de ese momento, se han utilizado diversos términos de acuerdo con la postulación de distintas clasificaciones y la terminología que utiliza la literatura para este defecto anatómico es variada: Busch (1897) lo nombró *dens in dente*; Rushton (1936) fue el primero en cambiar el término de Busch por odontoma dilatado compuesto; Hunter (1951) apoya el vocablo de Rushton y lo sugiere como el nombre adecuado; Oehlers (1957) se refiere a él como diente invaginado; Thomas (1974) lo llama *dens in dent*; Hulsmann (1997) lo identifica como guaridas en *dente*; Jung (2004) lo designa como diente telescópico, y Durack y Patel (2011) lo nombran dentoide en *dente*.

A través de algunos años, el término *odontoma dilatado* (OD) fue utilizado por algunos como parte de la clasificación de odontomas, en la que se encuentran el compuesto y el complejo, y se agrega al odontoma dilatado como una variante adicional; pero también, paralelamente, se comenzó a utilizar la denominación como sinónimo para referirse a la variante más compleja del DI.

A pesar de que los aspectos diagnósticos son claros, ambos términos se utilizan de manera constante como sinónimos, lo que genera en muchos casos confusión respecto del diagnóstico exacto y la definición. En la presente revisión, encontramos que el término OD es utilizado en casi la mitad de nuestras referencias.

Es preciso indicar que la definición y el origen de ambas entidades corresponden a procesos distintos, pues un diente invaginado es claramente una variante del desarrollo, mientras que la definición de un odontoma está contemplada como un crecimiento hamartomatoso.

Imagenológicamente, el DI (Clase III b) muestra una estructura radiopaca circundante, ya sea circular u ovalada, con un área radiolúcida central, y los incisivos laterales permanentes son los más afectados. Debido a la falta de signos clínicos, generalmente, los diagnósticos se efectúan mediante hallazgos radiológicos rutinarios en radiografías panorámicas; por este motivo, la tomografía computarizada de haz cónico (TCHC) debería ser considerada como un recurso complementario del diagnóstico.

El propósito de este estudio fue revisar el uso apropiado de la terminología utilizada y orientar mejor el conocimiento de una mejor definición para mejorar nuestros diagnósticos. Entender que la sinonimia puede ser motivo de identificación, sin dejar de lado el conocimiento de su origen.

## MATERIALES Y MÉTODOS

Para la realización de este artículo, se hizo una revisión literaria sistematizada de artículos referentes al tema publicados hasta el 15 de octubre de 2020, mediante las bases de datos Medline vía PubMed, SciELO y Scopus. También se consultaron diferentes revistas científicas de la salud como *Oral Surgery*, *Oral Medicine*, *Oral Pathology and Oral Radiology*, *Endodontics Journal*, *Journal Oral Maxillofacial Pathology*, *International Endodontic Journal*, *Oral Radiology* e *Iranian Endodontic Journal*. Las palabras claves utilizadas para esta investigación fueron odontoma, *dens in dente* y tomografía computarizada de haz cónico.

## DIENTE INVAGINADO (*DENS IN DENTE*)

El diente invaginado (DI) es una alteración en el desarrollo del órgano dentario, el cual se produce como consecuencia de una invaginación del epitelio interno del órgano del esmalte dentro de la papila dental, durante los estadios tempranos de la morfogénesis, antes de que el órgano dentario termine de mineralizarse; por lo regular, aparece como una apertura en la superficie coronal ^11^^,^^17^. Se presenta como una anomalía estructural del tejido dentario, en la cual se produce una dilatación de la corona hasta la porción de la raíz y, al darse la invaginación, compromete la cámara pulpar, antes de la calcificación de los tejidos dentales ^7^^,^^10^.

Se tiene conocimiento de una primera descripción de esta alteración en un diente de ballena y fue Ploquet (1794) quien detalló la forma que presentaba, que correspondía a una morfología de doble madriguera. Posteriormente, fue descrito como “un diente dentro de un diente” por Salter en 1855, y como *dens invaginatus* por primera vez en dientes humanos por un dentista de nombre Sócrates en 1856. John Tomes (1859) publicó el libro *A System of Dental Surgery*, en el que hace una descripción detallada de esta invaginación ^1^^,^^17^^,^^46^.

Entre los factores etiológicos se han propuesto algunas teorías, entre ellas una alteración del epitelio interno del órgano del esmalte, una disminución y nutrición inadecuada, un trauma, una fuerza o presión incontrolada producida en el germen dentario u otro mecanismo de presión, además de factores de origen genético ^7^^,^^15^^,^^17^^,^^18^^,^^21^. Existen teorías planteadas por distintos autores como Kronfel (1939), quien propone que es resultado del retraso en el crecimiento de una porción del epitelio del esmalte interno, mientras que el tejido restante continúa proliferando en dirección periférica; mientras que Bruszt (1950) menciona, en su Teoría Gemela, que el proceso mencionado es consecuencia de la fusión de dos gérmenes dentales. La infección localizada (Fischer, 1936; Sprawson, 1937) es considerada como factor causal, al igual que la presión de crecimiento que produce inestabilidad del germen dental (Euler, 1939; Atkinson, 1943) y los traumatismos ^11^^,^^12^.

Histológicamente, se observa que la invaginación coronaria está revestida de esmalte dentro de la papila dental; la pulpa expuesta, por lo general, produce inflamación y necrosis, mientras que la parte radicular está revestida de cemento y existe un pliegue de la vaina de Hertwig dentro de la raíz en desarrollo, lo que se asocia a lesiones periapicales ^11^^,^^14^; por ello, puede ser tubular o dilatada por debajo de un estrechamiento inicial de forma ampular como una cavidad de aspecto grande, por lo cual modifica en forma compleja esta patología, ya que todos los tejidos duros producen alteraciones macro y micro estructuradas ^11^^,^^13^^,^^15^. 

La prevalencia del diente invaginado es incierta; según su clasificación, tiene un rango estimado entre un 0,25% y un 10%, y se presenta con mayor frecuencia en la arcada superior, en los incisivos laterales superiores permanentes (85%), y es menos frecuente en incisivos centrales y, raramente, en dientes posteriores. No tiene predilección por sexo; sin embargo, algunos autores mencionan que es más frecuente en hombres que en mujeres, con una proporción de 3:1, y la ocurrencia bilateral se llega a presentar en un 43% de los casos sin importar si son dientes primarios, permanentes o supernumerarios ^12^^,^^15^^-^^18^^,^^36^.

Una primera clasificación del DI fue publicada por Hallett en 1953 y, posteriormente, Oehlers, en 1957, presenta otra en la que incluye 3 tipos según la profundidad de la invaginación y el grado de comunicación con el ligamento periodontal o el tejido periradicular ^47^. El tipo I, como una forma mínima de invaginación, con un saco ciego dentro de la corona que no sobrepasa la unión cemento-esmalte; el tipo II, en el que la invaginación sobrepasa la unión cemento-esmalte, se conserva dentro de la raíz y puede o no tener comunicación con la pulpa dental; y el tipo III, en el que la invaginación es más severa, ya que penetra y dilata la raíz del diente afectado, crea un doble foramen apical, la perfora y puede originar un segundo agujero en esta área o en la periodontal (tipo IIIa) y, además, se extiende apicalmente perforando el ápice (tipo IIIb); sin embargo, no hay comunicación directa con la pulpa. Un hecho curioso es que Oehlers, en su clasificación, menciona al tipo III con el sinónimo de odontoma dilatado, pero sugiere no utilizar este término ^8^^,^^9^^,^^12^^,^^14^^,^^15^^,^^18^^,^^21^^,^^37^^,^^47^.

## IMAGENOLOGÍA DEL DIENTE INVAGINADO

Existen diferencias imagenológicas que permiten diferenciar ambas alteraciones. Radiográficamente, el odontoma es radiopaco y se encuentra rodeado por una zona radiolúcida y otra corticalizada, bien definida. En los odontomas compuestos se observa una imagen radiopaca de densidad dentaria equivalente al número de dentículos presentes; en cambio, el odontoma complejo tiene el aspecto de una masa radiopaca bien delimitada la cual, ocasionalmente, puede estar ceñida por una zona radiolúcida estrecha ^21^^,^^25^. 

El aspecto radiológico de un DI depende de la severidad de la invaginación, ya que crea una zona radiolúcida con aspecto de asa en distintos tamaños hasta reflejar una imagen en forma de pera; esta última sería una invaginación severa en la que toma aspecto de un diente dentro de otro. Por su parte, en el tipo III, el diente se muestra totalmente deformado, con una forma circular u ovalada, y presenta una zona radiolúcida; su interior es radiotransparente y muestra una sola estructura con tejido blando en el centro, con una expansión abultada y tuberosa de la raíz del diente afectado. La valoración inicial con una radiografía periapical revela tanto el tipo, la extensión y la complejidad morfológica del DI como las pérdidas óseas reales ^3^^,^^8^^,^^11^^,^^14^^,^^22^.

Debido a que es una patología asintomática y de crecimiento lento, el DI suele ser diagnosticado por hallazgos radiográficos obtenidos mediante estudios panorámicos ^3^^,^^8^^,^^21^. La tomografía computarizada de haz cónico (TCHC) es considerada como la técnica radiológica más avanzada para el diagnóstico de esta malformación, ya que proporciona un enfoque más real en tres dimensiones de la invaginación y muestra datos radiológicos como dimensión, destrucción y oposición, además de la morfología interna anormal; por este motivo, se puede considerar que la TCHC es una herramienta más favorable para el diagnóstico y manejo ^11^^,^^24^. El objetivo principal de realizar estudios tridimensionales para diagnóstico imagenológico del DI es valorar el grado de invaginación, y de esta manera determinar el plan de tratamiento a seguir ^7^^,^^8^^,^^21^.

Debido a todas las variaciones en terminología existentes, el diagnóstico radiográfico permitirá distinguir el grado de invaginación mediante la diferencia de radiodensidades localizadas según la clasificación de Oehlers ^26^. Ya que no todas las imágenes permiten evaluar el tipo de malformación y que el diagnóstico de *dens in dente* suele ser accidental en radiografías convencionales que proporcionan una imagen bidimensional de una estructura tridimensional ^32^, el uso de la TCHC es recomendable porque permite evaluar la zona de la invaginación ^12^^,^^29^^,^^31^^,^^33^^-^^35^^,^^37^.

## DIENTE INVAGINADO U ODONTOMA DILATADO: ASPECTOS CONTROVERSIALES

En la revisión de la literatura, se mencionan diferentes términos para el DI, *dens in dente*, odontoma dilatado, odontoma invaginado, odontoma gestante, odontoma compuesto dilatado, diente incluido, dentoide *in dente*, guaridas *in dente*, diente telescópico, anomalía de la mujer embarazada y guaridas invaginatus ^26^^-^^30^. 

El término *odontoma dilatado* es el que genera confusión con relación al origen y la definición de la patología o alteración del desarrollo. El término odontoma pertenece a una clasificación determinada para una patología de tipo tumoral hamartomatosa y no para una alteración del desarrollo dental ^21^^,^^23^.

Rushton (1936) y Hunter (1951) han empleado *odontoma dilatado* para identificar el tipo III del diente invaginado ^27^. La diversidad de sinónimos empleada en esta anomalía provoca controversia. Hulsmann y Munir refieren que el término correcto sería *diente invaginado*, ya que es un despliegue de la porción externa (esmalte) en la porción interna (dentina), el cual forma una bolsa y un espacio muerto que se puede extender profundamente en la raíz ^18^. 

**Tabla 1:** Distribución de uso del término odontoma dilatado según frecuencia de referencias bibliográficas utilizadas

	Mencionan término odontoma dilatado	Mencionan solamente el término odontoma dilatado	Mencionan término diente invaginado
Referencias bibliográficas	21	11	42

Nota: Se utilizaron 47 referencias bibliográficas

Esta anomalía puede suceder tanto en la corona como en la raíz del diente durante su desarrollo ^23^^,^^26^^,^^31^ y se puede diferenciar clínica o radiológicamente por la zona en donde se presenta la invaginación: cuando su porción radicular está invaginada, se considera la forma más extrema y se denomina, erróneamente, *odontoma dilatado*. 

Otro aspecto a tener en cuenta es que este término se ha utilizado de manera oficial por muchos años, prueba de ello es que en la Clasificación Internacional de Enfermedades Aplicada a Odontología y Estomatología, de la OMS (1970), en el capítulo IX de Enfermedades del Aparato Digestivo en Enfermedades de la cavidad bucal, con el código 520.25, se clasifica como diente invaginado (sinónimo *dens in dente*, odontoma dilatado) y anomalías de los incisivos. Asimismo, hasta la tercera edición de esta clasificación, publicada en 1996 (pág. 63, código K00.25), lo clasifican como diente invaginado (*dens in dente*), odontoma dilatado y anomalías de incisivos.

El término OD también ha sido utilizado para describir al odontoma mismo. Broca (1867) definió e intentó por primera vez clasificar los odontomas según las etapas del desarrollo de los dientes. Thoma y Goldman (1946) realizaron una clasificación que incluye los siguientes términos: *odontomas compuestos germinados*, *odontomas compuestos*, *odontomas compuestos complejos*, *odontomas dilatados* (la corona o la parte de la raíz del diente muestra un marcado agrandamiento) y *odontomas quísticos*^40^. En ese sentido, es correcto indicar que la clasificación de la OMS mantiene a los odontomas entre las cuatro lesiones que contienen esmalte y dentina de apariencia normal ^25^. 

A pesar de que el diente invaginado tipo III suele ser denominado frecuentemente como *odontoma dilatado*, es importante tener claro que ambos términos se refieren a una deformidad dentaria que se presenta como una sola estructura calcificada que cuenta con una porción central más radiolúcida. 

La primera vez que se utilizó el término *odontoma dilatado* fue para describir un tipo de invaginación grave; sin embargo, según la clasificación de tumor de cabeza y cuello de la OMS, un odontoma es un tumor hamartomatoso ^20^^,^^30^. Incluso, en las publicaciones realizadas por esta organización sobre la clasificación de tumores odontogénicos en los años 1971, 1992, 2005 y 2017, no existe una referencia al OD, solo pueden hallarse las clasificaciones para los odontomas compuesto y complejo ^43^^-^^45^. Por estas razones, se considera que el término más adecuado para esta malformación es DI tipo III.

Hay que tomar en cuenta que el término *odontoma dilatado* se ha utilizado de manera errónea, ya que no entra como tal en ninguna clasificación de tumores ni en las anomalías de desarrollo dental.

Al realizar esta revisión de la literatura, se ha observado que aproximadamente el 50% de los autores citados se refieren a esta malformación como odontoma dilatado por costumbre, a pesar de que, actualmente, en la Clasificación de Tumores Odontogénicos de la Organización Mundial de la Salud no existe una entrada para *odontoma dilatado*. Por otro lado, y como parte de esta investigación, también se consultaron buscadores científicos como MeSh o DeCs, sin resultados para la búsqueda.

Queda claro que se trata de una misma entidad; sin embargo las controversias de esta denominación podrían seguir causando confusión y quizá las publicaciones científicas no deberían utilizarlo como definitorio, sino como una referencia histórica para corroborar el estudio de esta malformación.

## CONCLUSIONES

Existe una extensa controversia con respecto al uso del término *odontoma dilatado*, el mismo que se usa como sinónimo para identificar al diente invaginado tipo III. Algunos autores lo utilizaron en algún momento como definición de una variante de odontomas, lo que generó algunas confusiones diagnósticas. Sin embargo, por un proceso de derecho consuetudinario, con el paso del tiempo, el término OD se sigue utilizando para definir al diente invaginado.

Diversas clasificaciones han utilizado estos términos como sinónimos, incluso la OMS lo mencionaba en una de sus publicaciones. Actualmente, sigue siendo utilizado como sinónimo, por lo que es importante conocer su origen y tener claro que un odontoma (*per se*) es un tumor, mientras que un diente invaginado es una anomalía del desarrollo dentario, y que la controversia está basada en referencias históricas y de costumbre. 

## Fuente de financiamiento:

Autofinanciado
